# Skin collagen advanced glycation endproducts (AGEs) and the long-term progression of sub-clinical cardiovascular disease in type 1 diabetes

**DOI:** 10.1186/s12933-015-0266-4

**Published:** 2015-09-05

**Authors:** Vincent M Monnier, Wanjie Sun, Xiaoyu Gao, David R Sell, Patricia A Cleary, John M Lachin, Saul Genuth, The DCCT/EDIC Research Group

**Affiliations:** Departments of Pathology, Case Western Reserve University School of Medicine, Cleveland, OH USA; Departments of Biochemistry, Case Western Reserve University School of Medicine, Cleveland, OH USA; The Biostatistics Center, The George Washington University, Rockville, MD USA; Department of Medicine, Case Western Reserve University School of Medicine, Cleveland, OH USA; http://www.diabetes.niddk.nih.gov/dm/pubs/control/

**Keywords:** AGEs, A1c, DCCT/EDIC, Coronary artery calcium, Intima media thickening, Cardiac indices

## Abstract

**Background:**

We recently reported strong associations between eight skin collagen AGEs and two solubility markers from skin biopsies obtained at DCCT study closeout and the long-term progression of microvascular disease in EDIC, despite adjustment for mean glycemia. Herein we investigated the hypothesis that some of these AGEs (fluorescence to be reported elsewhere) correlate with long-term subclinical cardiovascular disease (CVD) measurements, i.e. coronary artery calcium score (CAC) at EDIC year 7–9 (n = 187), change of carotid intima-media thickness (IMT) from EDIC year 1 to year 6 and 12 (n = 127), and cardiac MRI outcomes at EDIC year 15–16 (n = 142).

**Methods:**

Skin collagen AGE measurements obtained from stored specimens were related to clinical data from the DCCT/EDIC using Spearman correlations and multivariable logistic regression analyses.

**Results:**

Spearman correlations showed furosine (early glycation) was associated with future mean CAC (p < 0.05) and CAC >0 (p = 0.39), but not with CAC score <100 vs. >100. Glucosepane and pentosidine crosslinks, methylglyoxal hydroimidazolones (MG-H1) and pepsin solubility (inversely) correlated with IMT change from year 1 to 6(all P < 0.05). Left ventricular (LV) mass (cMRI) correlated with MG-H1, and inversely with pepsin solubility (both p < 0.05), while the ratio LV mass/end diastolic volume correlated with furosine and MG-H1 (both p < 0.05), and highly with CML (p < 0.01). In multivariate analysis only furosine (p = 0.01) was associated with CAC. In contrast IMT was inversely associated with lower collagen pepsin solubility and positively with glucosepane,

**Conclusions:**

In type 1 diabetes, multiple AGEs are associated with IMT progression in spite of adjustment for A1c implying a likely participatory role of glycation and AGE mediated crosslinking on matrix accumulation in coronary arteries. This may also apply to functional cardiac MRI outcomes, especially left ventricular mass. In contrast, early glycation measured by furosine, but not AGEs, was associated with CAC score, implying hyperglycemia as a risk factor in calcium deposition perhaps via processes independent of glycation.

Trial registration: Registered at Clinical trial reg. nos. NCT00360815 and NCT00360893, http://www.clinicaltrials.gov

## Background

Patients with type 1 diabetes (T1DM) have a high risk of developing cardiovascular disease (CVD). The Diabetes Control and Complication Trial (DCCT)/Epidemiology of Diabetes Interventions and Complications (EDIC) has examined the impact of intensive glycemic treatment on the progression of subclinical CVD events in multiple studies. The findings indicate that coronary artery calcium deposition (CAC >) at EDIC year 7–9 was significantly lower in the intensive vs. conventional treatment group in the primary prevention but not secondary retinopathy intervention cohort. Waist to hip ratio, smoking, hypertension and hypercholesterolemia and HbA1c during DCCT and EDIC were also associated with the presence of CAC. Progression of carotid intima media thickness (IMT) at year 6 was significantly greater in the T1DM patients than in gender and age-matched controls and progression from EDIC year 1 to 6 was also lowered by intensive therapy. IMT was also associated with age, systolic blood pressure at EDIC onset, smoking, the LDL/HDL ratio, the urinary albumin excretion rate and mean HbA1c during the DCCT [1]. Finally, cardiac MRI studies performed at EDIC year 13–15 showed that greater left ventricular (LV) mass was associated with mean SBP, smoking and macroalbuminuria but not mean HbA1c over the entire preceding DCCT and EDIC period [2]. Mean A1c was however associated with the ratio of LV mass/EDV.

From another perspective, our previous research on the ability of skin advanced glycation products to predict complication progression in the DCCT/EDIC revealed a strong association between degree of skin collagen insolubilization and modifications by advanced glycation endproducts with the long-term progression rate and severity of microvascular disease [3–5]. In particular, addition of four skin collagen linked AGEs (i.e. glucosepane, carboxyethyl-lysine(CEL), methylglyoxal hydroimidazolone (MG-H1) and glyoxal hydroimidazolone (G-H1) to the original set of glycation products comprising furosine (glycated lysine a.k.a. fructose-lysine), carboxymethyl-lysine (CML), pentosidine, collagen fluorescence and collagen insolubility parameters significantly enhanced the strength of association between AGEs and the risk of progression over 13–16 years of microvascular and neuropathic complications during EDIC [6]. Multivariate logistic regression analysis showed that the glycation products glucosepane and furosine were most strongly associated with the risk of progression of retinopathy, furosine with nephropathy, and MG-H1 and furosine with clinical neuropathy. Most notably, adjustment for mean glycemia during the DCCT and EDIC did not impact on these associations, possibly implying a pathogenetic role of matrix bound AGEs for complications.

Based on the above findings we now investigated the association between the above skin glycation products and the progression of subclinical cardiovascular disease, namely coronary calcium deposition, carotid intima thickening and various parameters of diabetic cardiomyopathy in the same subset of the DCCT/EDIC cohort.

## Methods

### Subjects and outcomes

216 participants in the DCCT study from 8 clinics originally volunteered for a skin biopsy 1–2 years prior to DCCT closeout in 1993, as previously described [3]. Of these, 123 were from the primary cohort (1–5 years diabetes duration and no retinopathy on fundus photography) and 93 were in the secondary cohort (1–15 years diabetes duration and at least one retinal microaneurysm on fundus photography). Data on Coronary Artery Calcium (CAC) deposition measured by CT at EDIC years 7–9 [7] were available in 187 of those originally biopsied. Data on *carotid intima*–*media thickness* (IMT) measured by ultrasonography at EDIC year 6 and 12 [1] were available in 127 participants. The maximal IMT of the common carotid artery was defined as the mean of the maximal value for the near and far walls on both the right and left sides [1]. *Cardiac* magnetic resonance imaging (CMRI) *measures* were obtained in EDIC years 14–16 in 142 of the skin biopsy donors including left ventricular (LV) mass, (EDV) and end-systolic volume (ESV), stroke volume (SV), cardiac output (CO), ejection fraction (EF) and LV mass/EDV.

### Advanced glycation products (AGEs) in skin biopsies

Two skin biopsies were originally obtained at DCCT closeout in 1993, one of which was immediately processed for the measurement of 6 collagen solubility and glycation products, i.e. acid and pepsin solubility of collagen, collagen-linked fluorescence at 375 and 440 nm, furosine (acid conversion product of fructose-lysine) and the AGEs carboxymethyl-lysine (CML) and pentosidine, as described [3]. The second biopsy was stored under argon at −80 °C and processed in 2012 using enzymatic proteolysis to release the acid labile products including the AGE crosslink glucosepane together with those derived from the oxoaldehydes methylgloxal (MG-H1) and glyoxal (G-H1). These were assayed by liquid chromatography mass spectrometry as previously described [6]. The acid stable carboxyethyl-lysine (CEL) was included in this panel. Thus, analyses assessed the association of these nine markers with subclinical measures of macrovascular disease, whereby findings on collagen-linked fluorescence will be reported in a subsequent study addressing the molecular nature of fluorescence in type 1 diabetes.

### Statistical methods

Univariate differences between groups were assessed using Wilcoxon rank-sum test for quantitative or ordinal variables and Chi square test for categorical variables. Spearman correlations evaluated the association of skin collagen variables with other outcomes when adjusted for age and diabetes duration.

Since the level of CAC was not measurable for the majority of subjects, the associations with the quantitative levels of CAC were assessed using a Tobit regression model [8]. Associations with the quantitative levels of IMT and cMRI measures were assessed using normal errors linear models, that for IMT using an unstructured covariance structure [9].

Multivariable logistic regression models were assessed for the association of skin collagen AGE variables with the odds of abnormal levels of pre-clinical macrovascular measures. Significance of effects of specific covariate blocks, adjusted for other covariates, was evaluated using a likelihood ratio test (LRT) [10]. Backward elimination was used starting with all 10 AGEs (the original and new sets combined, including fluorescence) to select the subset of skin collagen variables nominally significantly associated with risk of complications at the 0.05 significance level. Entropy R^2^ was used to assess the strength of association in logistic regression models [10]. Odds ratio per standard deviation increase in the skin collagen AGE variables and HbA1c were reported. For univariate AGE effects, Benjamini and Hochberg’s false discovery rate (FDR) alpha adjustment for multiple tests was conducted to control the overall FDR at a level of 0.05 [11]. Unless otherwise noted, nominal p-values were presented. SAS software was used to perform these analyses.

## Results

### Clinical characteristics of participants

DCCT baseline characteristics of the 216 participants from the primary and secondary cohorts are summarized in Table 1. By design, the secondary cohort had longer diabetes duration and more advanced retinopathy [12]. The data show that it also had higher triglycerides and cholesterol, thicker intima media layers, higher CAC scores, but was not different in terms of cardiac subclinical endpoints (Table 2).

**Table 1:** Clinical characteristics of skin donors at DCCT baseline and mean AGE levels at time of DCCT biopsy

	Primary	Secondary	P value
N = 216	123	93	
DCCT baseline			
Female (%)	53 %	41 %	
Age (years)	27.1 (6.8)	29.6 (6.5)	0.009
Duration (months)	2.7 (1.4)	8.6 (4)	<0.001
HbA_1c_ (%) (mmol/mol)	9.1 (1.8)	8.9 (1.5)	
Mean blood glucose (mg/dl)	230.4 (82.8)	225.6 (71.5)	
Triglycerides (mg/dl)	72.4 (50.5)	92.9 (51.6)	<0.001
Cholesterol (mg/dl)	178.3 (32.7)	179.1 (36.5)	0.006
Retinopathy (%)			
10/10: None	100	0	
20/≤20: Microaneurysms only	0	60	
30/≤30: Mild NPDR	0	28	
45/≤45: Moderate NPDR	0	11	
AER >40 mg/24 h	1	10	0.003
Confirmed clinical neuropathy (%)	3	14	0.004
sBP (mmHg)	114.7 (11.9)	119.4 (10.5)	
dBP (mmHg)	72.9 (8.5)	75.1 (7.8)	
AGEs and solubility parameters			
Acid soluble (%)	0.5 (0.3)	0.6 (0.4)	
CEL (pmol/mg collagen)	141.4 (104)	152.5 (111.1)	
CML (pmol/mg collagen)	527.8 (140.4)	548.1 (119.2)	
Furosine (pmol/mg collagen)	756.2 (239.9)	786.2 (219.8)	
G-H1 (pmol/mg collagen)	66.8 (30.7)	63.6 (38)	
GSPNE (nmol/mg collagen)	2.5 (0.6)	2.6 (0.7)	
MG-H1 (nmol/mg collagen)	0.8 (0.4)	0.8 (0.5)	
Pentosidine (pmol/mg collagen)	25.3 (6.2)	26.6 (8.6)	
Pepsin Soluble (%)	6.8 (3.5)	6.5 (3)

**Table 2:** Subclinical cardiovascular outcomes of the skin biopsy study participants during EDIC

	Primary	Secondary	P value
Intima-media thickening study (N = 127)			
EDIC year 1	6 (0.7)	6.2 (0.8)	
EDIC year 6	6.2 (0.8)	6.5 (0.8)	0.003
EDIC year 12	6.8 (0.9)	7.1 (1.1)	
Coronary calcium study (N = 187)			
CAC Mean (Agatston units)	23 (109.2)	91.7 (281)	0.002
Cardiac MRI studies (N = 142)			
Left ventricular end diastolic mass (g)	146.6 (33.2)	149.6 (35.8)	
End diastolic volume (ml)	144.1 (34.4)	140.6 (31.6)	
End systolic volume (ml)	56.5 (16.3)	55.4 (17.7)	
Stroke volume (ml)	87.5 (21.7)	85.2 (19.4)	
Cardiac output (L/min)	6 (1.5)	5.9 (1.3)	
Ejection fraction (%)	60.8 (5)	61 (6.9)	
LV mass/end diastolic volume (g/ml)	1 (0.2)	1.1 (0.2)	

### Coronary artery calcium deposition

Of the 216 originally biopsied participants, 187 underwent computed tomography for measurement of CAC deposition analysis during EDIC years 7–9. Spearman correlation analysis reveals that furosine, i.e. the glucose-derived Amadori product significantly correlates with severity of coronary artery calcium deposition, both as quantitative variable (p = 0.026) as well as for the categorical comparison CAC >0 vs CAC = 0 (p = 0.039), though the effect was not significant for CAC >100 (Table 3).

**Table 3:** Associations of AGEs with sub-clinical cardiovascular disease outcomes

	N	Furosine	CML	Pentosidine	Pepsin soluble	Acid soluble	CEL	G-H1	MG-H1	Glucosepane
Coronary artery calcification										
CAC score (correlation coefficient, p value, FDR^#^)^^^	187	0.16* 0.026 (0.143)	0.12 0.09	0.12 0.11	−0.14 0.57	0.05 0.47	−0.08 0.26	0.11 0.11	0.04 0.55	0.07 0.33
CAC score = 0 vs. CAC score >0 (mean ± std)	131	749 (240)	528 (129)	25.3 (6.7)	6.7 (3.1)	0.5 (0.3)	151 (103)	61 (28)	0.8 (0.5)	2.6 (0.6)
	56	817 (230)	560 (148)	27.6 (8.5)	6.3 (2.5)	0.6 (0.4)	146 (124)	71 (38)	0.8 (0.5)	2.5 (0.7)
*P* Value (FDR)^+,#^		*0.039*	0.13	0.15	0.76	0.32	0.22	0.12	0.7	0.32
CAC score <100 vs. CAC score >100 (mean ± std)	170	766 (241)	535 (135)	25.8 (7.4)	6.6 (3.0)	0.5 (0.3)	148 (108)	64 (30)	0.8 (0.4)	2.6 (0.6)
	17	808 (223)	567 (142)	27.2 (6.3)	5.9 (2.7)	0.6 (0.6)	162 (130)	72 (41)	0.9 (0.5)	2.6 (0.7)
P-Value (FDR)^+,#^		0.32	0.25	0.38	0.27	0.73	0.87	0.49	0.29	0.65
Common IMT (correlation)										
Year 6	127	0.17	0.14	0.13	−0.11	0.02	0.10	−0.06	0.21*	0.19*
Change: year 6–year 1	127	0.04 0.68	0.04 0.20	0.23* 0.01	−*0.20* * 0.016	0.02 0.94	0.07 0.29	0.05 0.45	0.22* 0.059	0.18* 0.047
Year 12	127	0.15	0.06	−0.04	0.00	0.02	0.05	−0.02	0.06	0.16
Change: year 12–year 1	127	0.07	0.06	0.02	−0.13	−0.05	0.03	0.10	0.01	0.15
Cardiac MRI studies										
LV Mass (g)	142	0.10 0.25	0.16 0.058	0.14 0.11	−0.17* 0.04	−0.16 0.054	−0.02 0.83	0.03 0.75	0.17* 0.049	0.07 0.44
End diastolic volume (ml)	142	−0.05	−0.02	0.09	−0.10	−0.10	0.06	−0.12	0.00	−0.07
End systolic volume (ml)	142	−0.06	−0.02	0.07	−0.07	0.00	0.07	−0.06	0.01	−0.09
Stroke volume (ml)	142	−0.03	−0.03	0.07	−0.07	−0.12	0.04	−0.10	−0.03	−0.03
Cardiac (L/min)	142	0.14	0.02	0.06	−0.08	−0.08	0.01	−0.01	−0.08	0.05
Ejection fraction (%)	142	0.02	−0.05	−0.07	0.05	−0.07	−0.02	−0.04	−0.04	0.01
LV mass/end diastolic volume (g/ml)	142	0.18*	0.22**	0.08	−0.13	−0.13	−0.05	0.09	0.19*	0.15

Italic values indicate statistically significant associations

* p < 0.05, ** p < 0.01.

^^^Analyses of correlations and their associated p values used Spearman correlations.

^+^P values between categorical results were generated using the Wilcoxon two sample test.

^#^Numbers in parentheses are the Benjamini and Hochberg’s FDR alpha adjustment.

Multivariate regression analysis (Table 4) confirmed the association between quantitative CAC score with furosine and showed that it was weakened upon adjustment for DCCT mean A1c but not for EDIC mean A1c. Multivariate analysis also confirmed the Spearman correlation data showing that furosine was not associated with higher CAC scores (not shown). In contrast, the association between quantitative CAC score or CAC score >0 with DCCT mean A1c (p = 0.025; p = 0.031, respectively) became non-significant upon adjustment for furosine (Table 4), perhaps suggesting that at the tissue level is a more relevant risk factor than at the blood level. EDIC mean A1c was not associated with CAC score.

**Table 4:** Multivariable regressions of AGE effect on CT calcification outcomes (N = 187)

CAC score (quantitative)*			
Covariate effects from multiple models	X^2^	P value	Geometric mean ratio (95 % CI)^^^
Furosine:			
Unadjusted for A1c	5.7	*0.017*	5.6 (1.4, 22.7)
Adjusted for			
DCCT mean A1c	1.3	0.26	3.8 (0.4, 37.3)
EDIC mean A1c	4.4	*0.035*	5.0 (1.1, 22.2)
DCCT mean A1c:			
Unadjusted	5.0	*0.025*	1.9 (1.1, 3.3)
Adjusted for			
Furosine	0.2	0.68	1.2 (0.5, 3.0)
EDIC mean A1c:			
Unadjusted	0.6	0.52	1.1 (0.7, 1.8)

CAC >0**			
Covariate effects from multiple models	t value	P value	Odds ratio (95 % CI)^#^
DCCT mean A1c:			
Unadjusted	2.2	0.031	1.3 (1.0, 1.6)
EDIC mean A1c:			
Unadjusted	0.8	0.41	1.1 (0.9, 1.5)

CAC >100**			
Covariate effects from multiple models	t value	P value	Odds ratio (95 % CI)^#^
DCCT mean A1c:			
Unadjusted	0.4	0.68	1.1 (0.7, 1.6)
EDIC mean A1c:			
Unadjusted	0	0.71	1.1 (0.6, 2.0)

Italic values indicate statistically significant associations

* Models for CAC mean score were based on a Tobit-censored regression which evaluated the effect of AGE or HbA1c on the outcome of log (CT score)-log (lowest detectable CT score) allowing for unmeasurable values below the lower limit of quantification. Both unadjusted and adjusted models were adjusted for scanning site, baseline age, diabetes duration, sex, and DCCT baseline retinopathy cohort (primary vs. secondary). Adjusted models further included both AGE and HbA1c as risk factors.

** Models for CAC >0 and CAC >100 were based on a Generalized Linear Mixed Model (GLIMMIX) logistic regression which evaluated the effect of AGE or HbA1c on the prevalence of CAC >0 or CAC >100. Both unadjusted and adjusted models were adjusted for baseline age, diabetes duration, sex, and DCCT baseline retinopathy cohort (primary vs. secondary) with scanning site as a random effect. Adjusted models further included both AGE and HbA1c as risk factors

^^^Geometric mean ratio of CAC scores was the ratio of predicted CAC scores for a one unit increase in HbA1c.

^#^Odds ratio was the ratio of odds for CAC > 0 or CAC > 100 based on a one unit increase in HbA1c.

### Intima media thickness (IMT)

Data were available in 127 participants. Spearman correlation analysis (Table 3) showed that the progression of IMT between EDIC year 1 and 6 was positively associated with pentosidine, methylglyoxal hydroimidazolone and glucosepane, but inversely associated with collagen pepsin solubility (all p values <0.05). Multivariate regression analysis (Table 5) confirmed these associations and revealed that adjustment for DCCT and EDIC mean A1c did not abolish the association with MG-H1 and pentosidine, though GSPN became non-significant. Collagen solubility by pepsin was inversely associated with IMT (p = 0.048) and the association was abolished by adjustment with mean A1c. Mean DCCT and EDIC A1c were not associated with IMT in this skin biopsy cohort, although DCCT mean A1c was associated with IMT in the complete DCCT cohort [1]. Most of the associations between the collagen modification and IMT vanished beyond year 6.

**Table 5:** Multivariable regressions of AGE effect on IMT outcomes (N = 127)

IMT change from year 1 to year 6			
Covariate effects from multiple models*	t value	P value	LSMean difference (95 % CI)^^^
MG-H1			
Unadjusted	2.6	*0.012*	0.01 (0.00, 0.02)
Adjusted for			
DCCT mean A1c	2.5	*0.014*	0.01 (0.00, 0.02)
EDIC mean A1c	2.7	*0.008*	0.01 (0.00, 0.02)
Pentosidine			
Unadjusted	2.4	*0.016*	0.01 (0.00, 0.03)
Adjusted for			
DCCT mean A1c	2.4	*0.020*	0.02 (0.00, 0.03)
EDIC mean A1c	2.3	*0.022*	0.01 (0.00, 0.03)
Glucosepane			
Unadjusted	2.0	*0.048*	0.01 (0.00, 0.02)
Adjusted for			
DCCT mean A1c	0.7	0.48	0.00 (−0.01, 0.01)
EDIC mean A1c	−0.4	0.67	−0.00 (−0.01, 0.01)
Pepsin soluble			
Unadjusted	−2.0	*0.048*	−0.03 (−0.06, -0.00)
Adjusted for			
DCCT mean A1c	−1.9	0.06	−0.03 (−0.07, 0.00)
EDIC mean A1c	−1.7	0.08	−0.03 (−0.06, 0.00)
DCCT mean A1c:			
Unadjusted	0.7	0.48	0.02 (−0.01, 0.01)
EDIC mean A1c:			
Unadjusted	1.5	0.14	0.05 (−0.00, 0.02)

Italic values indicate statistically significant associations

* Models were based on a general linear regression which evaluated the effect of respective AGE or HbA1c on the change of IMT from EDIC year 1 to year 6. Both unadjusted and adjusted models were adjusted for IMT levels at EDIC year 1. Adjusted models further included both the respective AGE and HbA1c as risk factors.

^^^LSMean difference was the difference of estimated Least Square mean of IMT change from EDIC year 1 to year 6 based on a one standard deviation increase in AGE (Pentosidine: 7.3, Pepsin soluble: 6.7, MG-H1: 0.44, GSPNE: 0.66: or a one unit increase in HbA1c.

### Leftventricular thickness and other cardiac functional parameters (cMRI)

Among the various cardiac indices measured by cMRI between EDIC year 14 and 16, left ventricular mass (LV) was significantly associated with skin modifications, in particular furosine, pepsin solubility (inversely), and MG-H1 (Table 3). As for the entire DCCT cohort [2], mean A1c values were not associated with LV mass.

In summary, except for the early glycation product furosine > DCCT mean A1c, none of the AGEs were associated with CAC. Progression of IMT was most strongly associated with MG-H1~pentosidine>glucosepane~pepsin insolubility based of the p values of the multivariate analysis. Finally, leftventricular mass was most strongly associated with MG-H1 and pepsin insolubility, while the LV mass over end diastolic volume ratio was most strongly associate with CML ≫ MG-H~furosine. These latter associations were found in Spearman but not multivariate regression analysis.

## Discussion

### Historical considerations

In a recent study we have shown that expansion of the original set of 6–10 skin collagen AGEs and parameters of collagen solubility enhances the association between the skin markers of long-term glycemic damage and the progression of microvascular complications [5]. Notably the full set of all 10 AGE markers, including fluorescence, was associated with the risk of retinopathy, nephropathy and neuropathy progression in spite of adjustments for mean DCCT and EDIC A1c. Collagen glycation, i.e. fructose-lysine measured as furosine acid conversion product, was most consistently associated with either past or future progression of all three microvascular complications in EDIC. Adjustment for DCCT A1c did not nullify these robust associations except for nephropathy. Similarly, the associations between glucosepane and retinopathy, and MG-H1 with neuropathy were not nullified by adjustment for DCCT A1c, respectively. Finally, the association between DCCT mean A1c and each microvascular complication was nullified by adjustment for AGEs, as a group or individually. The above results raised the compelling question of the extent to which skin AGEs measured up to 19 years prior to assessment of subclinical indicators of macrovascular disease were also associated with their progression in type 1 diabetes, and whether specific AGEs were associated with specific subclinical macrovascular disease.

### Associations between early glycation, advanced glycation and CAC deposition

In this study, only furosine (early glycation) and mean DCCT A1c were associated with CAC. This latter association was nullified upon adjustment for furosine indicating a close relationship between glycemia at the tissue level and the process leading to calcium deposition in the coronary arteries of individuals with type 1 diabetes. In view of the fact that none of the specific advanced glycation end products predicted this association, it is difficult to envisage a direct mechanistic role in this process. However, van Eupen et al., found that plasma AGEs are associated with CAC deposition [13]. In that regard CML was found to accelerate calcification in diabetic rat, possibly by engaging RAGE in macrophages and induction of the BMP-2-cbfα1-ALP-calcification cascade [14]. However, since the association uncovered by van Eupen et al. was preferentially with pentosidine, a marker of increased ascorbic acid oxidation [15], a more likely explanation is that CAC is accelerated by hyperglycemia mediated oxidant stress and NADPH oxidase activity which triggers BMP activation and calcium deposition [15].

Possibly implicating indirect effects in which CML-rich proteins e.g. stimulate BMP deposition [16] into the arterial wall.

### Association with intima-media thickness

In contrast to coronary artery calcium deposition, a causal relationship between IMT and glycation is strongly suggested from the observation that IMT is associated with multiple AGE crosslinks, such as glucosepane and pentosidine, as well as methylglyoxal hydroimidazolone MG-H1. Methylglyoxal itself is a source of methylglyoxal-derived imidazole (MODIC) crosslinks which, although not measured here, are also elevated in skin collagen from diabetic individuals [17]. Together, these three arginine-lysine AGE crosslinks may participate in decreasing collagen solubility, explaining the significant inverse relationship noted between pepsin solubility and IMT change between year 1 and 6. A role for glucosepane crosslinks in this process is strongly supported by the observation that glucosepane is the single major AGE and crosslink in skin collagen from individuals with diabetes [17]. Moreover, the Oslo Diabetes Study recently reported similar associations between skin glucosepane and both IMT and pulse wave velocity in 27 participants with type 1 diabetes of very long duration [18].

Somewhat puzzling is that the association between AGEs and IMT became non-significant at year 12, though both furosine and glucosepane were borderline significant reinforcing the potential role of matrix crosslinking in IMT. One explanation is the contribution of lipid abnormalities became with time less important as determinant of IMT progression as that the number of participants on statins increased from 2.94 to 13.2 and 49 % at EDIC year 1, 6 and 12, respectively. In addition, glycemia improved in the control group with mean A1c values decreasing concomitantly from 8.9 to 8.6 and 8.3 %, respectively. These two metabolic effects likely altered either the IMT progression rate and/or the collagen turnover rate, resulting in weakened ability for skin AGEs to predict the very long-term IMT progression rate.

### Association with left ventricular mass and LV mass/end diastolic volume ratio

A similar relationship between collagen solubility and LV mass was observed, although neither furosine nor glucosepane were themselves associated with LV mass. This may suggest that cardiac hypertrophy is only indirectly influenced by hyperglycemia, perhaps via methylglyoxal levels, as suggested by the association between MG-H1 and LV mass. The observation that the ratio of LV mass/end diastolic volume was associated with furosine and MG-H1, as well as the glycoxidation product CML, is surprising because CML was not associated with any of the other subclinical complications. In contrast, numerous studies showed that serum or plasma CML is strongly associated with cardiovascular disease in diabetes and aging [19–21]. The potential mechanism and implication of these findings is that CML formation from fructose-lysine could be catalyzed by Cu^2+^ [22]. Indeed Cu^2+^ chelation was shown to ameliorate diabetic cardiomyopathy [23], thus supporting the proposed association between abnormal copper metabolism and diabetic cardiomyopathy [24].

### Potential role of methylglyoxal in subclinical CVD complications

The observation that MG-H1 is associated with IMT independently of glycemia, LV mass and LV mass/EDV raises the question of whether methylglyoxal plays a mechanistic role in the pathogenesis of atherosclerotic complications. This proposition is supported by the lower levels of MG-H1 (and glucosepane!) in EDIC subjects with prior DCCT intensive control [5] and the findings of lower IMT levels in the intensive treatment group of the whole DCCT/EDIC cohort [1]. A direct role for methylglyoxal lysine dimer (MOLD) [25] or MODIC [17] crosslinks has been alluded to, though these are present in amounts 100 times lower than glucosepane in skin from diabetic individuals [17]. Blocking of arginine residues by MG-H1 together with crosslinking might contribute to lowering collagen turnover and thickening of the artery wall. Indeed Chong et al. found the methylglyoxal modified collagen binds less well phagocytes thought to play a role in collagen turnover [26]. However, there is also the possibility that higher methylglyoxal levels could stimulate collagen deposition. Several authors noted that cells incubated with methylglyoxal modified collagen induced collagen expression, TGF β1 expression [27] and promoted myofibroblast differentiation [28]. Methylglyoxal also exerts it’s toxicity by inhibiting cell binding to the modified matrix [29] resulting in anoikis [30]. Finally, a profibrotic effect was directly demonstrated when methylglyoxal given orally to mice lead to kidney collagen accumulation [31].

Finally, as noted above, this study’s findings parallel the recently observed glucosepane –IMT association in the Oslo study [18], and also the similar recent observation by the same investigators of an association between impaired left ventricular dysfunction, MG-H1 and glyoxal hydroimidazolone G-H1, with the caveat that MG-H1 was measured in the serum and G-H1 in skin [32]. Taken together, they raise the important question of whether a combination of glycation burden and altered cell signalling by the glycated extracellular matrix is the elusive mediator in the sclerotic process that underlies the pathogenesis of accelerated cardiovascular disease in diabetes.

### Limitations in the current study

The major limitation of our study is that the demonstrated associations between skin AGEs and subclinical CVD outcomes cannot prove a cause and effect relationship. Moreover, none of the many specific skin AGE molecules correlated consistently with any specific CAC, carotid IMT or CMRI outcome, undoubtedly reflecting the complex pathogenesis of CVD in T1DM. Nonetheless, the false discovery rate was held to 0.05, minimizing the possibility that these are chance findings resulting from the multiple statistical tests that were conducted. Moreover, the positive association between plasma pentosidine and CAC has been found in T1DM [13] supports the proposition that AGEs are possibly involved in the development of CAC in individuals with T1DM. Similarly, specific AGE combinations, notably pentosidine and CEL, were strongly associated with micro- and macrovascular complications in the Medalist Study [33]. Thus, these data are in keeping with the present study findings and reinforce them, even though p values were not as strong as for microvascular disease outcomes [5]. Yet, in spite of these important findings, a major practical limitation is that biopsies for risk biomarker assessment are unlikely to be routinely implemented, and that therefore non-invasive methods will be needed to assay specific AGEs in skin.

## Conclusions

These findings in a substantial DCCT/EDIC cohort add strong support to the premise that glycation of proteins and formation of crosslinks in the extracellular matrix likely plays a significant role in the pathogenesis of macrovascular complications in T1DM, especially what concerns the thickening of the carotid intima-media. Similar reasoning may apply to the enlargement of the left ventricle. However, coronary artery calcium deposition, while associated with collagen glycation per se, does not appear to be mediated by any specific matrix-linked AGEs. This latter conclusion might have to be revised in the future if a higher number of events helps uncover associations with specific AGEs.

## Abbreviations

A1c: hemoglobin A1c
AGE: advanced glycation endproduct
CAC: coronary artery calcium
CML: N^e^-carboxymethyl-l-lysine
cMRI: cardiac magnetic resonance imaging
CVD: cardiovascular disease
DCCT: Diabetes Complications and Control Trial
EDIC: Epidemiology of diabetes Interventions and Complications
EDV: end-diastolic volume
ESV: end-systolic volume
FDR: false discovery rate
G-H1: glyoxal hydroimidazolone- 1
IMT: intima media thickness
LV: left ventricular
LW-1: long wave fluorophore 1
MG-H1: methyglyoxal hydroimidazolone
MODIC: methyl glyoxal dimer imidazolone crosslink
MOLD: Methylglyoxal lysine dimer
T1DM: type 1 diabetes mellitus
SIF: skin intrinsic fluorescence
