# Erratum to: Skin collagen advanced glycation endproducts (AGEs) and the long-term progression of sub-clinical cardiovascular disease in type 1 diabetes

**DOI:** 10.1186/s12933-015-0295-z

**Published:** 2015-10-09

**Authors:** Vincent M Monnier, Wanjie Sun, Xiaoyu Gao, David R Sell, Patricia A Cleary, John M Lachin, Saul Genuth, The DCCT/EDIC Research Group

**Affiliations:** Departments of Pathology, Case Western Reserve University School of Medicine, Cleveland, OH USA; Departments of Biochemistry, Case Western Reserve University School of Medicine, Cleveland, OH USA; The Biostatistics Center, The George Washington University, Rockville, MD USA; Department of Medicine, Case Western Reserve University School of Medicine, Cleveland, OH USA; http://www.diabetes.niddk.nih.gov/dm/pubs/control/

## Erratum to: Cardiovasc Diabetol (2015) 14:118 DOI 10.1186/s12933-015-0266-4

After publication of the original article it came to the authors’ attention that there was an error in the ‘Results’ section of the abstract. The value ‘0.39’ should have been given as ‘0.039’. This error has now been corrected in the original article.

